# Comparison of Efficacy among Three Radiofrequency Ablation Techniques for Treating Knee Osteoarthritis: A Systematic Review and Meta-Analysis

**DOI:** 10.3390/ijerph18147424

**Published:** 2021-07-12

**Authors:** Shih-Hsiang Chou, Po-Chih Shen, Cheng-Chang Lu, Zi-Miao Liu, Yin-Chun Tien, Peng-Ju Huang, Cheng-Ming Chou, Chia-Lung Shih

**Affiliations:** 1 Department of Orthopedics, Kaohsiung Medical University Hospital, Kaohsiung 807, Taiwan; stanelychou@gmail.com (S.-H.C.); shenporch@gmail.com (P.-C.S.); cclu0880330@gmail.com (C.-C.L.); d740113@kmu.edu.tw (Y.-C.T.); roger01@ms4.hinet.net (P.-J.H.); 2 Orthopaedic Research Center, Kaohsiung Medical University, Kaohsiung 807, Taiwan; 3 Department of Orthopedics, Kaohsiung Municipal Siaogang Hospital, Kaohsiung 812, Taiwan; 4 College of Medicine, Kaohsiung Medical University, Kaohsiung 807, Taiwan; p21actin@yahoo.com.tw; 5 Department of Orthopedics, Ditmanson Medical Foundation Chia-Yi Christian Hospital, Chia-Yi City 600, Taiwan; 6 Clinical Medicine Research Center, Ditmanson Medical Foundation Chia-Yi Christian Hospital, Chia-Yi City 600, Taiwan

**Keywords:** cooled radiofrequency, conventional radiofrequency, pulsed radiofrequency, knee osteoarthritis

## Abstract

Radiofrequency ablation (RFA) was first introduced for treating knee osteoarthritis (OA) in 2010 and has emerged as a minimally invasive treatment option. Three RFA techniques have been adopted for treating knee OA, including conventional, pulsed, and cooled RFA. However, the efficacy among different RFA techniques in the treatment of knee OA is still unclear. Three electronic databases were systematically searched for relevant articles, including PubMed, Embase, and Cochrane Library. A meta-analysis of articles that investigated the use of RFA techniques in the treatment of knee OA was conducted to pool the effect size in pain before and after treatment. A total of 20 eligible articles (including 605 patients) were included for our meta-analysis. After treatment, the patients had significant improvements in pain for all three RFA techniques when compared with the baseline level for the 1, 3-, and 6-month follow-ups (*p* < 0.00001). However, there were no significant differences in the efficacy among the three RFA techniques for all follow-up visits (*p* > 0.05). The three RFA techniques demonstrated a significant improvement in pain for up to 6 months after treatment. Comparing the efficacy of the three RFA techniques in the treatment of knee OA, our results showed that no significant differences in pain relief among the three RFA techniques were observed at the 1-, 3-, 6, and 12-month follow-up visits.

## 1. Introduction

Knee osteoarthritis (OA) is a degenerative disease and is one of the main causes of disability and pain worldwide. Pain and disability can reduce quality of life, such as social connectedness, emotional well-being, and relationships [1]. It was reported that at least 19% of American adults (age ≥ 45 years) suffer from this disease [2]. To delay or avoid surgical treatment, several non-surgical treatment options have been used to treat knee OA, such as extracorporeal shockwave therapy [3], intra-articular hyaluronic acid [4], intra-articular platelet-rich plasma [5], and foot orthoses [6].

Radiofrequency ablation (RFA) was first introduced for treating knee OA in 2010 [7] and has emerged as a minimally invasive treatment option [8]. Three RFA techniques have been adopted for treating knee OA, including conventional [7], pulsed [9], and cooled RFA [8]. However, the mechanism of action of the three RFA techniques is different, and thus their efficacy in the treatment for knee OA might be dissimilar.

A previous meta-analysis has reported that the use of RFA for treating knee OA could provide short-term improvement in joint function recovery for 3 months and pain relief for 6 months [10]. To date, only a single review has compared the efficacy among the three RFA techniques for the treatment of knee OA [11]. However, their results could not support the superiority of any specific RFA techniques, and they did not conduct a meta-analysis to draw these results [11]. The efficacy of the three RFA techniques for treating knee OA has not been well compared using a meta-analysis. Although these three RFA techniques have been used to treat knee OA for a long time, it is important to provide clinical evidence for comparing their efficacy, so the objective of this study was to compare their efficacy in the treatment of knee OA using a meta-analysis. We expected that this study could provide valuable information in choosing an appropriate RFA technique with the best efficacy.

## 2. Materials and Methods

### 2.1. Search Strategy

We followed the Preferred Reporting Items for Systematic Reviews and Meta-Analyses guidance to conduct this study. Three electronic databases were independently searched by two authors (S.-H. C. and C.-L. S.) for relevant articles from inception to 12 February 2020, including PubMed, Embase, and Cochrane library. The following key word combinations were adopted for searching relevant articles: (“*radiofrequency ablation*” OR “*radiofrequency*”) AND (“*knee osteoarthritis*” OR “*knee arthritis*”). The detailed search process from the three databases is shown in Table S1. Firstly, related articles were identified from the three databases using the key word combinations. Then, duplicates were removed, and the remaining articles were screened by title/abstract analysis. Finally, the possible articles related to our topic were screened by full-text analysis, and any differences were discussed until a consensus was achieved. In addition, the references from included studies and review articles related to our topic were manually searched for extra articles.

### 2.2. Eligibility Criteria

The inclusion criteria were as follows: (1) articles investigating the efficacy of pulsed, conventional, or cooled RFA technique; (2) patients with knee OA; (3) visual analog scale (VAS) or numeric rating scale (NRS) used to evaluate pain level; and (4) articles written in English or Chinese. The exclusion criteria were as follows: (1) animal studies; (2) editorials; (3) letters; (4) reviews; (5) conference abstracts; (6) case reports; and (7) no outcomes regarding VAS or NRS scores.

### 2.3. Types of Outcomes

Two measures (VAS and NRS) were adopted in this study to assess the efficacy of RFA in the treatment of knee OA pain. VAS and NRS are self-reported measures for assessing pain level. The range of both measures are from 0 to 10, in which 0 indicates no pain and 10 indicates the worst pain. Therefore, VAS and NRS can be taken as the same measure when conducting our meta-analysis.

### 2.4. Data Extraction

The main characteristics of included articles were extracted, including the first author’s name, publication year, type of RFA techniques, sample size, mean age of patients, measures of outcomes, and follow-up periods. If we could not extract detailed outcomes (mean and SD) from these articles, we tried to obtain the information by contacting authors via e-mail.

### 2.5. Quality Assessment

The methodological quality for each article was assessed using the Newcastle–Ottawa Scale (NOS) for cohort studies, which includes selection, comparability, and outcome components [12]. A NOS score of 1–3 indicates low quality, 4–6 indicates medium quality, and 7–9 indicates high quality.

### 2.6. Statistical Analysis

For subgroup analysis, these data were sub-grouped based on RFA techniques and follow-up periods. The follow-up periods were grouped into 1 month (3–4 weeks), 3 months, 6 months, and 12 months. Standardized mean difference (SMD) and 95% confidence interval (CI) were used to assess the effect size in pain before and after treatment. Heterogeneity among articles was assessed using *I*^2^ statistic and *X*^2^ test. When *I*^2^ statistic > 50% or a *p*-value of *X*^2^ test < 0.05, the outcomes among articles indicated heterogeneity and a random-effects model was used to assess the effect size; otherwise, a fixed-effects model was adopted. We used the Review Manager software package (version 5.4; Cochrane collaboration) to conduct these statistical analyses. To assess the effect of factors (imaging method or operation time) on efficacy of RFA, meta-regression analysis was performed. Meta-regression analysis was conducted using Comprehensive Meta-Analysis V2 Software.

### 2.7. Ethical Approval

Human ethical approval was not required because no issues regarding participant privacy were adopted in this study.

## 3. Results

### 3.1. Literature Review

**Figure 1:**
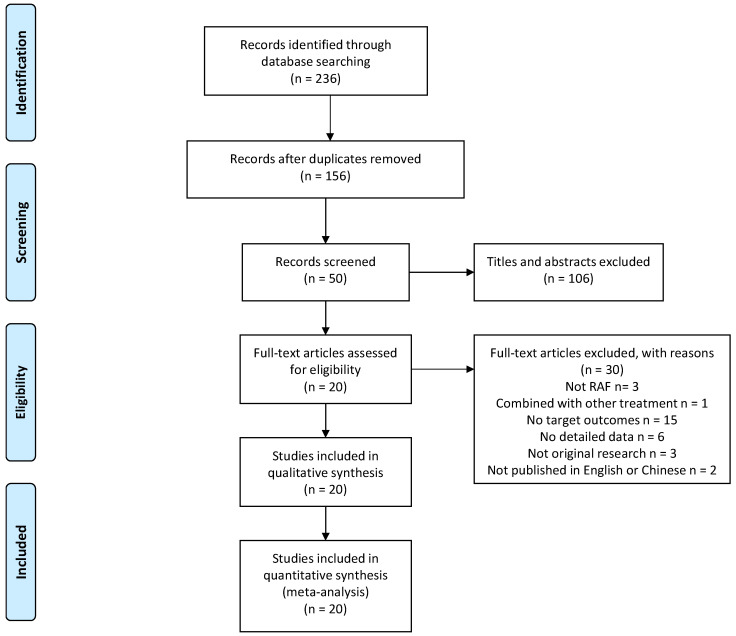
Preferred Reporting Items for Systematic Reviews and Meta-Analyses flowchart of systematic review.

After initial review of the three databases, a total of 236 records were retrieved, including 58 from PubMed, 91 from Embase, and 87 from Cochrane library (Figure 1). A total of 80 duplicates were identified and removed from further analysis, and the remaining 156 articles were screened through title or abstract. In total, 50 records met our inclusion/exclusion criteria. These articles were screened by full-text analysis, and 30 records were excluded with reasons. Three articles did not use RFA to treat knee OA. One article combined with other treatment. Fifteen articles did not report the target outcomes. Six records did not provide detailed outcomes. Three records were not original research. Two records were not published in English or Chinese. Finally, a total of 20 eligible articles were included in our meta-analysis [7,9,13,14,15,16,17,18,19,20,21,22,23,24,25,26,27,28,29,30].

### 3.2. Main Characteristics

These 20 articles (that included 605 patients) were published from 2011 to 2019 (Table 1). One of them was published in Chinese, and the others were all published in English. The number of trials treated by conventional, pulsed, and cooled RFA techniques were 11, 8, and 2, respectively. For conventional RFA, 8 of 11 trials reported that genicular nerves were subjected to RFA. One reported peripheral nerves, and two did not record which nerves were subjected to RFA. For pulsed RFA, only three trials reported which nerves were subjected to RFA and they were genicular nerves, genicular nerves, and composite nerves, respectively. However, the trials using cooled RFA did not report which nerves were subjected to RFA. Sixteen trials recorded which imaging method was adopted and most of them used ultrasound or fluoroscopy. All these patients suffered from knee OA pain and the severity of this disease ranged from grades I to IV according to the Kellgren–Lawrence classification. The mean age of these patients ranged from 53.3 to 77.2 years. Sixteen articles used VAS, and the other four articles used NRS to assess patient pain levels. The follow-up period of extracted outcome ranged from 3 weeks to 12 months.

### 3.3. Quality Assessment

The quality of each article was assessed using the NOS. The patients were primary knee OA for all articles. However, we did not consider a control group in our meta-analysis for all articles. The knee OA patients were diagnosed according to radiographical examination for all articles. All of the articles did not demonstrate outcome of interest at start of study. We did not consider a control group in our meta-analysis and the comparability could not be assessed. All the articles used self-reported measures to assess outcome. All the articles had long follow-up periods (≥3 weeks) for outcomes to occur. Lost-to-follow-up rates were small (<20%) for all articles except for two articles [9,13]. After assessing the quality, the scores of these articles ranged from 4 to 5. All of them showed medium quality (score of 4–6) (Table 2).

### 3.4. Meta-Analysis

(1) One-month follow up

**Figure 2:**
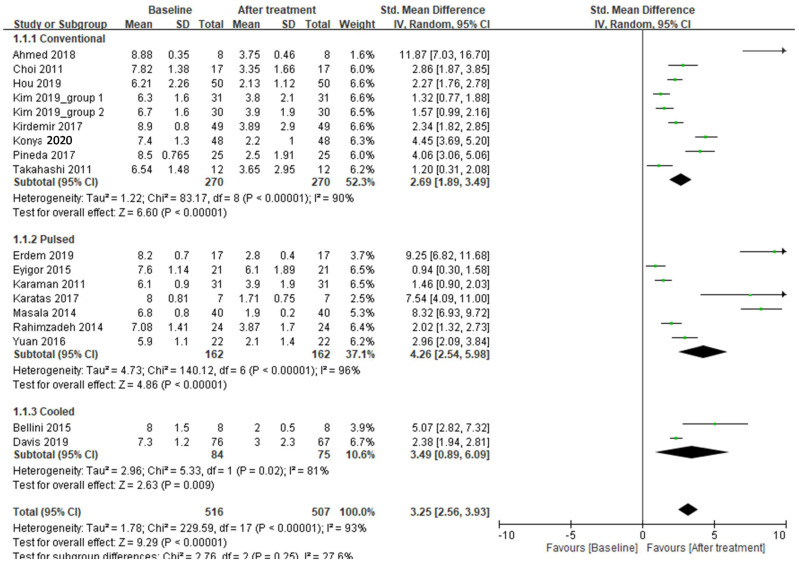
Meta-analysis of efficacy in pain using three radiofrequency ablation techniques to treat knee OA at the 1-month follow-up.

A total of nine trials that used conventional RFA in the treatment of knee OA recorded pain scores at the 1-month follow-up. These results demonstrated heterogeneity (*I*^2^ = 90%) among studies (Table 1), and the random-effects model was used to pool the results. The patients had significant improvement in pain after treatment (random-effects model: nine trials, SMD = 2.69, 95% CI = 1.89 to 3.49, *p* < 0.00001 for 1-month vs. baseline) (Figure 2). A total of seven trials that adopted pulsed RFA for treating knee OA recorded pain scores at the 1-month follow-up. These results demonstrated heterogeneity (*I*^2^ = 96%) among studies (Figure 2), and the random-effects model was used to pool the results. The result demonstrated that the patients had significant improvement in pain after treatment (random-effects model: seven trials, SMD = 4.26, 95% CI = 2.54 to 5.98, *p* < 0.00001 for 1-month vs. baseline) (Figure 2). Only two trials that used cooled RFA for treating knee OK recorded pain scores at 1-month follow-up. These results demonstrated heterogeneity (*I*^2^ = 81%) among studies (Figure 2), and the random-effects model was used. Significant improvement in pain was observed after treatment (random-effects model: two trials, SMD = 3.49, 95% CI = 0.89 to 6.09, *p* = 0.009 for 1-month vs. baseline) (Figure 2). When comparing the efficacy among the three RFA techniques, no significant differences in pain relief were observed (*p* = 0.25) (Figure 2).

(2) Three-month follow-up

**Figure 3:**
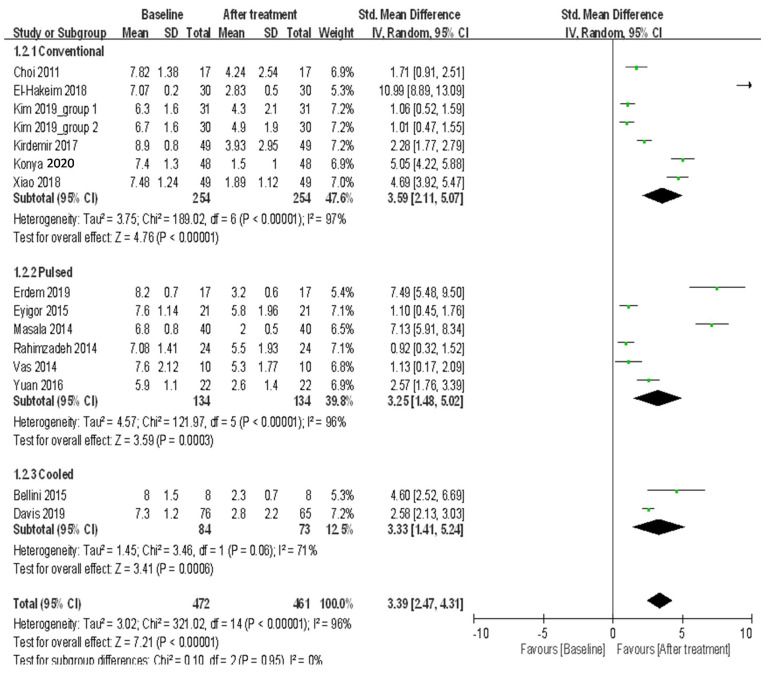
Meta-analysis of efficacy in pain using three radiofrequency ablation techniques to treat knee OA at the 3-month follow-up.

A total of seven trials that used conventional RAF for treating knee OA recorded pain scores at the 3-month follow-up. These results demonstrated heterogeneity (*I*^2^ = 97%) among studies (Figure 3), and the random-effects model was used to pool the results. The result demonstrated that the patients had significant improvement in pain after treatment (random-effects model: seven trials, SMD = 3.59, 95% CI = 2.11 to 5.07, *p* < 0.00001 for 3-month vs. baseline) (Figure 3). A total of six trials that used pulsed RFA for treating knee OA recorded pain scores at the 3-month follow-up. These results demonstrated heterogeneity (*I*^2^ = 96%) among studies (Figure 3), and the random-effects model was used. The patients had significant improvement in pain after treatment (random-effects model: six trials, SMD = 3.25, 95% CI = 1.48 to 5.02, *p* = 0.0003 for 3-month vs. baseline) (Figure 3). Two trials that used cooled RFA for treating knee OK recorded pain scores at the 3-month follow-up. These results demonstrated heterogeneity (*I*^2^ = 71%) among studies (Figure 3), and the random-effects model was used. Significant improvement in pain was observed after treatment (random-effects model: two trials, SMD = 3.33, 95% CI = 1.41 to 5.25, *p* = 0.0006 for 3-month vs. baseline) (Figure 3). When comparing the efficacy among the three RFA techniques, no significant differences in pain relief were observed (*p* = 0.95) (Figure 3).

(3) Six-month follow-up

**Figure 4:**
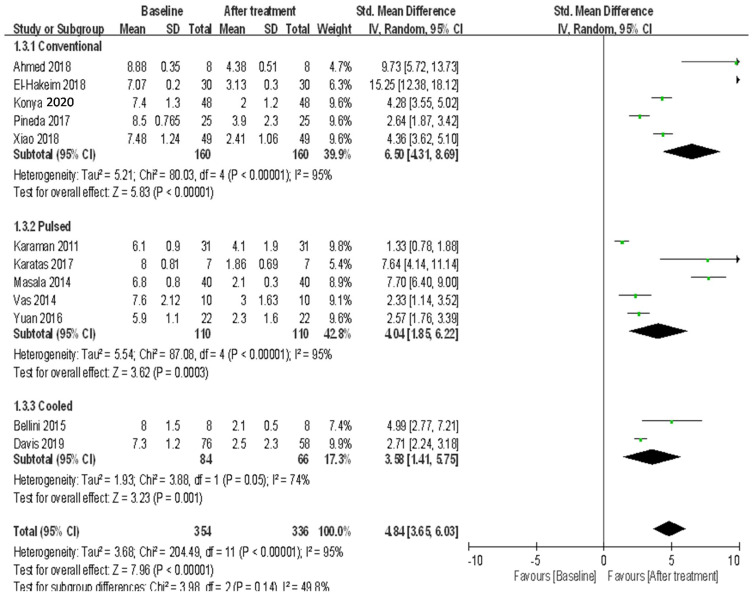
Meta-analysis of efficacy in pain using three radiofrequency ablation techniques to treat knee OA at the 6-month follow-up.

A total of five trials that used conventional RAF for treating knee OA recoded pain scores at the 6-month follow-up. These results demonstrated heterogeneity (*I*^2^ = 95%) among studies (Figure 4), and the random-effects model was used. The patients had significant improvement after treatment (random-effects model: five trials, SMD = 6.50, 95% CI = 4.31 to 8.69, *p* < 0.00001 for 6-month vs. baseline) (Figure 4). A total of five trials that used pulsed RAF for treating knee OA recoded pain scores at the 6-month follow-up. These results demonstrated heterogeneity (*I*^2^ = 95%) among studies (Figure 4), and the random-effects model was used. The patients demonstrated significant improvement in pain after treatment (random-effects model: five trials, SMD = 4.04, 95% CI = 1.85 to 6.22, *p* = 0.0003 for 6-month vs. baseline) (Figure 4). Two trials that used cooled RFA for treating knee OA recorded pain scores at the 6-month follow-up. These results showed heterogeneity (*I*^2^ = 74%) among studies (Figure 4), and the random-effects model was used. The patients demonstrated significant improvement in pain after treatment (random-effects model: two trials, SMD = 3.58, 95% CI = 1.41 to 5.75, *p* = 0.001 for 6-month vs. baseline). When comparing the efficacy among the three RFA techniques, no significant differences in pain relief were observed (*p* = 0.14) (Figure 4).

(4) 12-month follow-up

**Figure 5:**
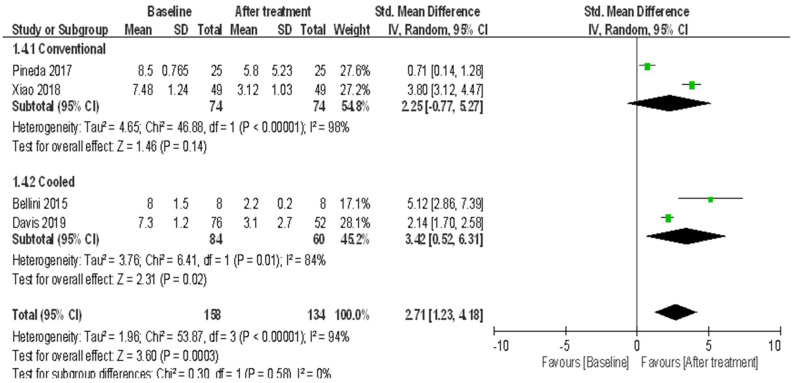
Meta-analysis of efficacy in pain using two radiofrequency ablation techniques to treat knee OA at the 12-month follow-up.

Only two trials conducting conventional RAF for treating knee OA recorded pain scores at the 12-month follow-up. These results demonstrated heterogeneity (*I*^2^ = 98%) among studies (Figure 5), and the random-effects model was used. The patients showed significant improvement in pain after treatment (random-effects model: two trials, SMD = 2.25, 95% CI = −0.77 to 5.27, *p* = 0.14 for 12-month vs. baseline) (Figure 5). Only two trials conducting cooled RF for treating knee OA recorded pain scores at the 12-month follow-up. These results showed heterogeneity (*I*^2^ = 84%) between studies (Figure 5), and the random-effects model was adopted. The patients showed significant improvement in pain after treatment (random-effects: two trials, SMD = 3.42, 95% CI = 0.52 to 6.31, *p* = 0.02 for 12-month vs. baseline) (Figure 5). The efficacy between two RFA techniques did not show significant difference (*p* = 0.58) (Figure 5).

### 3.5. Meta-Regression

To assess the effect of factors on the efficacy of RFA in the treatment of knee OA, meta-regression was performed. In the process of RFA treatment, we found that imaging method and application time were regularly recorded in the articles using conventional RFA. The results demonstrated that neither imaging method nor operation would affect the efficacy of conventional RFA at 1-, 3-, or 6-month follow-up-visits, respectively (Table 3).

### 3.6. Adverse Effects

Among 10 articles using conventional RFA, eight recorded adverse effects after treat-ment [7,13,15,17,18,19,20,21]. All of them reported that no serious adverse effects or complications related to conventional RFA were observed. Among eight articles using pulsed RFA, five recorded adverse effects after treatment [9,24,25,26,28]. They all reported that no serious adverse effects or complications related to pulsed RFA were observed. Among two articles using cooled RFA, only one recorded adverse effects after treatment [29]. No serious adverse effects or complications were reported after receiving cooled RFA.

## 4. Discussion

Radiofrequency ablation has been used to treat knee OA pain for over a decade [31]. Although different RFA techniques have been developed to treat knee OA, the efficacy of these RFA techniques has not been well compared. In this study, we conducted a meta-analysis to compare the efficacy among different RFA techniques in the treatment of knee OA. The results showed that all the three RFA treatment groups had significant improvements in knee OA pain relief for all follow-up visits compared with the baseline level. However, no significant differences in efficacy among the three RFA techniques were observed for all the follow-up visits.

The mechanisms of action of the three RFA techniques are different. Pulsed RFA was designed to avoid damaging neuronal tissue. Thus, pulsed RFA uses lower temperature and less energy when compared with conventional RFA. Cooled RFA is a relatively new technique and was designed to increase lesion size [32]. This implies that the efficacy of the three RFA techniques in the treatment of knee OA may be different. We conducted a meta-analysis to compare the efficacy among the three RFA techniques in the treatment of knee OA pain. However, our results showed that the efficacy of the three RFA techniques for treating knee OA pain had no significant difference among them.

To compare the efficacy among different treatments using a meta-analysis, trials that compare different treatments are usually considered, such as randomized controlled trials [33,34]. Results derived from meta-analyses of this type are thought to have a high level of evidence; however, a limited number of randomized controlled trials comparing different RFA techniques in the treatment of knee OA have been published. Thus, it is difficult to compare the efficacy among different RFA techniques for treating knee OA using a meta-analysis [11], but the efficacy in change of clinical outcome from baseline to follow-up visits was adopted in this study. The use of meta-analysis to compare the efficacy among treatments by comparing the efficacy in differences from baseline to follow-up visits has been adopted in a previous meta-analysis [35]. We believe that the results of our meta-analysis calculated by this method were reliable.

Due to nerve regeneration after RFA treatment, its efficacy in pain relief would decrease with time [28]. However, we did not find this event in our meta-analysis for any of the three RFA techniques. The possible reason may be that the outcomes of these articles show heterogeneity for all subgroup analyses and the efficacy estimated from these analyses may not be precise. Moreover, the long-term outcomes (>12 months) of the three RFA techniques were not provided in this study. The efficacy of long-term outcomes of the three techniques is still controversial [16,20,26,29,30]. This implies that nerve regeneration may occur after 12 months, and we could not observe this event from our results.

This meta-analysis has some limitations, which may affect our conclusions. Firstly, the methodology used to compare the efficacy among different techniques was estimated from the differences in pain scores between baseline and follow-up visits, so these results might lack a high level of evidence (such as randomized controlled trials). More high-quality randomized controlled trials should be conducted to compare the efficacy among different RFA techniques in future research to provide a more robust level of evidence. Secondly, the three RFA techniques did not provide standard treatment procedures in the treatment of knee OA. This might produce differences in efficacy among different articles when using the same RFA technique; thus, our results may not be accurate. Thirdly, joint function is one of the primary outcomes to assess the efficacy of knee OA. There are only a limited number of articles reporting clinical outcomes regarding joint function, and we could not compare the efficacy in joint function among different RFA techniques; thus, the efficacy in joint function among the three RFA techniques is still unknown. Finally, our meta-analysis included a limited number of articles that included cooled RFA (*n* = 2), and our conclusions might not be reliable when comparing cooled RFA.

## 5. Conclusions

Our results showed that there were no significant differences in pain relief among the three RFA techniques. However, our meta-analysis used the improvement in pain between baseline and follow-up visits to compare the efficacy among the three RFA techniques, and such subjective results lack a high level of evidence. More randomized controlled trials should be further conducted to confirm our results.

## Figures and Tables

**Table 1:** Main characteristics of the articles included in this study.

OBS	Author And Publication Year	Type of Radiofrequency	Target Nerve	Imaging Method	Sample Size	Mean Age (Year)	Sex (M/F)	K–L Grade	Type of Outcome	Follow-Up Time	Brief Result
1	Kim 2019_group 1 [13]	Conventional	genicular nerve	ultrasound	31	65.2	9/22	II–IV	NRS	1 and 3 months	No significant difference in NRS between ultrasound- vs. fluoroscopy-guided RFA.
	Kim 2019_group 2 [13]	Conventional	genicular nerve	fluoroscopy	30	66.8	8/22	II–IV			
2	Hou 2019 [14]	Conventional	NA	meridians-muscles theory	50	64	15/35	NA	VAS	1 month	The efficacy of RFA was better than celecoxib capsules
3	Konya 2020 [15]	Conventional	genicular nerve	fluoroscopy	48	77.2	19/29	III–IV	VAS	1, 3, and 6 months	RFA was effective and safe treatment option.
4	Xiao 2018 [16]	Conventional	peripheral nerve	NA	49	56.5	12/37	NA	VAS	3, 6, and 12 months	RFA was more effective than sodium hyaluronate injection
5	El-Hakeim 2018 [17]	Conventional	genicular nerve	fluoroscopy	30	62	9/12	III–IV	VAS	3 and 6 months	RFA could relieve pain and disability.
6	Ahmed 2018 [18]	Conventional	genicular nerve	ultrasound	8	61.5	5/3	III–IV	NRS	1 and 6 months	RFA was an effective treatment option.
7	Kirdemir 2017 [19]	Conventional	genicular nerve	fluoroscopy	49	64	8/41	II–IV	VAS	1 and 3 months	RFA led to significant pain reduction and functional improvement.
8	Pineda 2017 [20]	Conventional	genicular nerve	ultrasound	25	75	3/22	III–IV	VAS	1, 6, and 12 months	RFA could alleviate intractable pain and disability.
9	Choi 2011 [7]	Conventional	genicular nerve	fluoroscopy	17	67.9	2/15	II–IV	VAS	1 and 3 months	RFA led to significant pain reduction and functional improvement.
10	Takahashi 2011 [21]	Conventional	NA	fluoroscopy	12	70.4	1/10	II–VI	VAS	3 weeks	RFA provided a remarkable pain relief effect.
11	Erdem 2019 [9]	Pulsed	genicular nerve	ultrasound	17	69.8	5/12	III–IV	VAS	3 weeks and 3 months	RFA could significantly alleviate pain and disability.
12	Karatas 2017 [22]	Pulsed	NA	NA	7	54.4	NA	II–III	VAS	1 and 6 months	RFA could be successfully used. in the treatment of pain.
13	Yuan 2016 [23]	Pulsed	NA	NA	22	69.9	7/15	NA	VAS	1, 3, and 6 months	RFA could alleviate the clinical symptoms and decrease the content of TNF-α, MMP-3 and IL-1 in the synovial.
14	Eyigor 2015 [24]	Pulsed	NA	NA	21	61.2	NA	NA	VAS	1 and 3 months	RFA was effective and safe for the pain treatment.
15	Rahimzadeh 2014 [25]	Pulsed	NA	fluoroscopy	24	57.0	11/13	I–III	VAS	1 and 3 months	Intra-articular prolotherapy with erythropoietin was more effective than RFA in pain relief.
16	Masala 2014 [26]	Pulsed	pericapsular nerve	fluoroscopy	40	NA	22/18	III–IV	VAS	1, 3, and 6 months	RFA was effective for patients who were unresponsive. to conservative therapies.
17	Vas 2014 [27]	Pulsed	composite nerve	ultrasound	10	53.3	1/9	I-IV	NRS	3 and 6 months	RAF was a safe, effective, and minimally invasive technique.
18	Karaman 2011 [28]	Pulsed	NA	NA	31	62.8	9/22	NA	VAS	1 and 6 months	RAF was an effective and safe method.
19	Davis 2019 [29]	Cooled	NA	fluoroscopy	76	NA	NA	NA	NRS	1, 3, 6, and 12 months	RAF for pain could last for at least 12 months.
20	Bellini 2015 [30]	Cooled	NA	fluoroscopy	8	72	2/6	NA	VAS	1, 3, 6, and 12 months	RFA could improve pain and function for 12 months.

K–L: Kellgren–Lawrence; NRS: numeric rating scale; VAS: visual analog scale; NA: not available; RFA: radiofrequency ablation.

**Table 2:** Assessment of methodological quality by the Newcastle–Ottawa quality assessment scale for cohort studies.

Author	Selection				Comparability	Outcome			Total
	1	2	3	4	1	1	2	3	
Kim 2019 [13]	1	0	1	1	0	0	1	0	4
Hou 2019 [14]	1	0	1	1	0	0	1	1	5
Konya 2020 [15]	1	0	1	1	0	0	1	1	5
Xiao 2018 [16]	1	0	1	1	0	0	1	1	5
El-Hakeim 2018 [17]	1	0	1	1	0	0	1	1	5
Ahmed 2018 [18]	1	0	1	1	0	0	1	1	5
Kirdemir 2017 [19]	1	0	1	1	0	0	1	1	5
Pineda 2017 [20]	1	0	1	1	0	0	1	1	5
Choi 2011 [7]	1	0	1	1	0	0	1	1	5
Takahashi 2011 [21]	1	0	1	1	0	0	1	1	5
Erdem 2019 [9]	1	0	1	1	0	0	1	1	5
Karatas 2017 [22]	1	0	1	1	0	0	1	0	4
Yuan 2016 [23]	1	0	1	1	0	0	1	1	5
Eyigor 2015 [24]	1	0	1	1	0	0	1	1	5
Rahimzadeh 2014 [25]	1	0	1	1	0	0	1	1	5
Masala 2014 [26]	1	0	1	1	0	0	1	1	5
Vas 2014 [27]	1	0	1	1	0	0	1	1	5
Karaman 2011 [28]	1	0	1	1	0	0	1	1	5
Davis 2019 [29]	1	0	1	1	0	0	1	1	5
Bellini 2015 [30]	1	0	1	1	0	0	1	1	5

**Table 3:** Factors that may affect the improvement of conventional RFA were investigated by meta-regression analysis.

Factors	Β	SE	95% CI	*p*-Value
1-month follow-up				
Operation time	−0.00065	0.00042	−0.00147–0.00017	0.121
Fluoroscopy (Ref: Ultrasound)	−1.095	1.562	−5.035–1.086	0.206
3-month follow-up				
Fluoroscopy (Ref: Ultrasound)	3.062	3.501	−3.800–9.924	0.382
6-month follow-up				
Operation time	−0.018	0.023	−0.063–0.027	0.432
Fluoroscopy (Ref: Ultrasound)	3.420	7.055	−10.407–17.247	0.628

SE: standard error; CI: confidence interval; Ref: reference.

## Data Availability

All datasets generated for this research are included in this published article.

## Supplementary Materials

The following are available online at https://www.mdpi.com/article/10.3390/ijerph18147424/s1, Table S1: Search strategy.
