# Supplementary material for: Comparison of Efficacy among Three Radiofrequency Ablation Techniques for Treating Knee Osteoarthritis: A Systematic Review and Meta-Analysis
Source: Int J Environ Res Public Health. 2021 Jul 12;18(14):7424. doi: 10.3390/ijerph18147424 (PMC8306337; doi:10.3390/ijerph18147424)
Supplement: Supplementary file 1 [file ijerph-18-07424-s001.zip › ijerph-1189278-supplementary.pdf]

Table S1. Search strategy.

Database	Search details
Pubmed	((radiofrequency ablation) OR (radiofrequency)) AND ((knee osteoarthritis) OR (knee arthritis)) “Title/Abstract“
Embase	((radiofrequency ablation) OR (radiofrequency)) AND ((knee osteoarthritis) OR (knee arthritis)) “Title/Abstract“
Cochrane Library	((radiofrequency ablation) OR (radiofrequency)) AND ((knee osteoarthritis) OR (knee arthritis)) “Title/Abstract/Keywords“
